# Epidermal Growth Factor Receptor T790M Mutation Testing in Non-Small Cell Lung Cancer: An International Collaborative Study to Assess Molecular EGFR T790M Testing in Liquid Biopsy

**DOI:** 10.3390/cancers15133528

**Published:** 2023-07-07

**Authors:** Martin Filipits, Verena Kainz, Viktor Sebek, Herwig Zach, on behalf of the Liquid Biopsy Collaborative Study Group

**Affiliations:** 1 Center for Cancer Research, Medical University of Vienna, 1090 Vienna, Austria; 2 Division of Transplantation, Department of General Surgery, Medical University Vienna, 1090 Vienna, Austria; verena.kainz@meduniwien.ac.at; 3 Department of Pharmacology, Faculty of Medicine and Dentistry, Palacky University Olomouc, Hnevotinska st. 976/3, 779 00 Olomouc, Czech Republic; sebek.viktor@gmail.com; 4 Boehringer Ingelheim RCV GmbH & Co KG, 1120 Vienna, Austria; herwig.zach@boehringer-ingelheim.com

**Keywords:** liquid biopsy, EGFR, NSCLC, T790M mutation, NGS, PCR

## Abstract

**Simple Summary:**

Several liquid biopsy platforms with varying degrees of accuracy are available for EGFR mutation testing in NSCLC. We performed a collaborative study to describe and compare the sensitivity of different liquid biopsy platforms used in clinical routines to detect selected EGFR mutations. In-house PCR-based and NGS-based methods were used accordingly, and results were reported as in routine clinical practice. The results might offer an essential contribution to ensuring high-quality standards and contribute to developing a standard EGFR T790M testing in liquid biopsy.

**Abstract:**

Background: The detection of the EGFR T790M (T790M) mutation in non-small cell lung cancer (NSCLC) patients who progressed under treatment with first- or second-generation EGFR-tyrosine kinase inhibitors (TKIs) is important to offer a subsequent therapy with a third-generation EGFR-TKI. Liquid biopsy is a powerful tool to determine the T790M mutation status. Several liquid biopsy platforms with varying degrees of accuracy are available to test for T790M mutations, and sensitivities may differ among these methods. Methods: As no standard exists for the testing of T790M mutation in liquid biopsy, we performed a collaborative study to describe and compare the sensitivity of different in-house liquid biopsy platforms for the detection of the T790M mutation, EGFR exon 19 deletion (del19) and EGFR L858R mutation (L858R) across multiple participating laboratories in seven Central and Eastern European countries. Results: Of the 25 invited laboratories across Central and Eastern Europe, 21 centers participated and received 10 plasma samples spiked with cell-line DNA containing the T790M, del19, or L858R mutation in different concentrations. In-house PCR-based and NGS-based methods were used accordingly, and results were reported as in routine clinical practice. Two laboratories, which used the AmoyDx^®^ EGFR 29 Mutations Detection Kit (AmoyDx) with Cobas^®^ cfDNA Sample Preparation Kit and QX200 Droplet Digital PCR (ddPCR) with the QIAamp Circulating Nucleic Acid Kit identified all ten samples correctly. Cobas^®^ EGFR Mutation Test v2 (Cobas), the NGS methods, and the Idylla^TM^ detection method used in this study performed within the known sensitivity range of each detection method. Conclusions: If a negative result was obtained from methods with lower sensitivity (e.g., Cobas), repeated liquid biopsy testing and/or tissue biopsy analysis should be performed whenever possible, to identify T790M-positive patients to allow them to receive the optimal second-line treatment with a third-generation EGFR TKI.

## 1. Introduction

Epidermal growth factor receptor (EGFR) mutations are common oncogenic alterations in patients with non-small-cell lung cancer (NSCLC). Activating somatic deletions in exon 19 (del19) and the single amino acid substitution L858R in exon 21 are the “classical” EGFR mutations, accounting for more than 85% of all EGFR mutations in NSCLC patients [1]. The activating mutations of the EGFR genes in NSCLC lead to constitutive tyrosine kinase activity. Tyrosine kinase inhibitors (TKIs) were designed to bind the ATP-binding site of the EGFR kinase domain, thereby inhibiting its activity [2].

EGFR TKIs of the first- or second-generation, such as gefitinib, erlotinib, afatinib, icotinib, and dacomitinib, demonstrated higher overall response rate (ORR) and longer progression-free survival (PFS) compared with chemotherapy and are therefore standard first-line treatments for EGFR-positive NSCLC [1,3]. Response rates are between 56% to 74%, with a median progression-free survival of about twelve months [4]. However, patients treated with first- and second-generation EGFR TKIs often develop acquired resistance and disease progression due to a substitution of threonine with methionine at position 790 in exon 20, also known as T790M mutation [5]. Several studies demonstrated a T790M-positivity rate between 66% and 74% in EGFR-TKI pretreated NSCLC patients, depending on the method used [4,6,7,8]. A better understanding of the acquired resistance mechanism led to the development of third-generation EGFR-TKIs such as osimertinib, which are active against exon 19 and 21 mutations as well as the T790M mutation.

Osimertinib was the first FDA and EMA-approved third-generation EGFR-TKI which received marketing authorization in the first-line for patients with advanced tumors harboring activating EGFR mutations and for patients whose cancer cells show T790M mutations after prior EGFR-TKI treatments [3,9]. Osimertinib is an irreversible, oral TKI that demonstrated prolonged PFS compared to chemotherapy as a second-line treatment and was associated with higher response rates in patients with NSCLC harboring a T790M mutation [10,11,12,13]. These results led to establishing of osimertinib as the standard of care second-line treatment for EGFR T790M mutation-positive NSCLC [14,15]. The correct re-characterization of the tumor is therefore crucial for initiating subsequent therapy for NSCLC patients who progressed under EGFR-TKI therapy. Detecting EGFR mutations in tumor tissue samples is sometimes difficult when insufficient tumor material is available and/or poor patient performance status or other clinical limitations do not allow invasive procedures.

A liquid biopsy is a powerful option for the molecular characterization of the patient’s tumor status by minimal-invasively collecting circulating tumor cells (CTCs) or circulating tumor cellular components from the plasma of patients [16,17,18]. Cell-free circulating tumor DNA (ctDNA) are highly fragmented genomic pieces resulting from tumor cell apoptosis, necrosis, or the secretion of extracellular vesicles [19]. ctDNA forms a tiny fraction (<1%) of total cell-free DNA and several technologies have been developed to detect EGFR mutations, including amplified refractory mutation system (ARMS), digital droplet polymerase chain reaction (ddPCR), and next-generation sequencing (NGS). Commercially available kits, such as Roche Cobas^®^ EGFR mutation test v1 and v2 (Cobas), use the allele-specific ARMS method to detect up to 1–5% of mutant DNA. On the other hand, ddPCR and BEAMing (Beads, Emulsions, Amplification, and Magnetics) combine digital PCR and flow cytometry and allow the detection of DNA to a threshold of 0.01–0.03%. NGS can analyze key genes or screen the entire tumor genome for detecting novel mutations; the sensitivity may vary between NGS platforms, and exceptionally sensitive NGS-based methods have been described as able to detect mutant DNA in the presence of 10,000 wild-type DNA molecules [20].

Liquid biopsy can be used to identify T790M mutations even if the tumor tissue is not available, thereby maximizing the number of patients who may benefit from subsequent treatment with osimertinib in the case of T790M-positivity [21]; indeed, analyses of blood samples from 119 patients who had progressed on first- or second-generation EGFR-TKI revealed a plasma T790M-positivity of 71%. Minimal invasive plasma genotyping represents an attractive alternative for detecting T790M, and cell-free plasma DNA analysis can replace tissue analysis in selected cases [4]. Previous studies showed that ddPCR detected a substantially higher number of T790M mutations in plasma samples than Cobas [6]. Moreover, in a cross-platform comparison of different technologies for detecting the T790M mutation, the digital platforms outperformed the non-digital platforms [22]. It has been shown that digital PCR is clinically useful for selecting patients who had progressed during first-line EGFR-TKI therapy for the treatment with osimertinib [4].

Since no quality standards for detecting EGFR alterations using liquid biopsy exist, different pathological laboratories use their own protocols and platforms of choice depending on individual factors, including turnaround time or cost. Therefore, the sensitivity of the methods can vary greatly among different institutions. However, an accurate testing strategy is needed to identify all patients with T790M correctly so that patients can receive appropriate subsequent therapy. An inter-laboratory comparison of the individual liquid biopsy test strategies can provide insight into the performance of different methods currently used in routine clinical practice.

The aim of this collaborative study was to evaluate the routine clinical use of liquid biopsy at different pathological laboratories in Central and Eastern Europe and to describe the possible differences in the sensitivity of the methods used. These results may contribute to the development of standardization in liquid biopsy testing of EGFR T790M mutations and lead to the identification of more T790M mutation-positive NSCLC patients after progression in need of a targeted subsequent therapy.

## 2. Material and Methods

### 2.1. Participants

For feasibility reasons, a first testing wave was conducted in selected laboratories between May and July 2020. A second testing wave occurred between May and August 2021, involving more laboratories in seven Central and Eastern European countries. All the laboratories remained anonymous. The laboratories used their own in-house methods of choice for the preparation and detection of the T790M mutation in the first testing wave and the detection of T790M, del19, and L858R in the second testing wave.

### 2.2. Sample Panel Composition and Distribution

**Figure 1:**
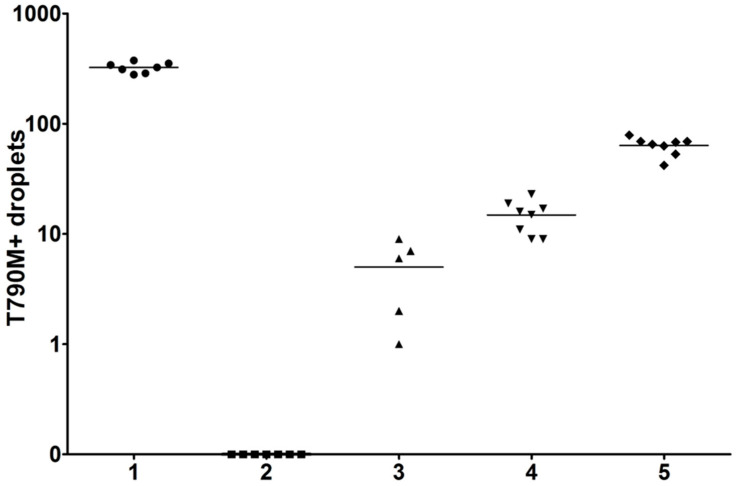
Different dilutions of the HCC-8207 cell line DNA included in the first testing wave of the collaborative study. The mean number of T790M-positive droplets (5–8 independent measurements) was 324 in sample 1, 0 in sample 2, 5 in sample 3, 17 in sample 4, and 72 in sample 5.

The sample panels were prepared in the Center for Cancer Research, Medical University of Vienna, and shipped to each participant laboratory. In the first testing wave of the collaborative study, sets of five cell line DNA samples (0.1 mL) were prepared at different dilutions. Each set consisted of four positive samples and one negative sample. The entire sample was to be used only for EGFR T790M testing, as determined. Details of the sample composition are shown in Figure 1.

In the second testing wave of this collaborative study, sets of ten plasma samples with a total volume of 1 mL each were sent to each laboratory and should be tested for T790M, del19, and L858R mutations. The cell lines used for spiking the plasma were HCC-8207 (T790M, del19), CRL-5908 (T790M, L858R), A549, and MCF-7 for EGFR wild-type (wt), respectively (Table 1). DNA was isolated from cell lines using the DNeasy Blood & Tissues kit (Qiagen, Hilden, Germany) according to the manufacturer’s instructions. Each plasma was spiked accordingly and contained various dilutions, except the negative control (sample No 3, 8). Samples No. 1 and 6 contained very high copy numbers of T790M plus del19, and 790M plus L858R, respectively, and should be detectable with any method. Samples No. 3 and 8 served as negative controls, and sample No. 2 was the challenging one with the fewest positive droplets (5 droplets for T790M and 7 for L858R). Three samples (sample No. 4, 7, 9) were at the threshold for Cobas with 25 to 34 copies and two samples (No. 5, 10) with an intermediate range of droplets.

Information about the collaborative study and specific instructions for using the samples and reporting the results were sent via e-mail to each laboratory 2–3 weeks in advance to allow sufficient time to answer any remaining questions about the procedure. All samples were coded to blind the assay, shipped frozen on dry ice to each laboratory, and consisted of five (first testing wave) or ten samples (second testing wave) at various dilutions.

## 3. Results

A first testing wave was carried out to establish the procedure. In this first wave, twelve diagnostic laboratories from Austria (n = 5), Bulgaria (n = 1), Lithuania (n = 1), Poland (n = 4), and Switzerland (n = 1) participated in the collaborative study (Table 2A). In the second wave, 21 laboratories from Austria (n = 9), Bulgaria (n = 1), Croatia (n = 1), Hungary (n = 3), Lithuania (n = 1), Poland (n = 2), and Slovakia (n = 4) joined the test (Table 2B).

A summary of the methods used by the laboratories for DNA analytics is shown in Table 3 for each wave. The method used was left to the laboratories, and test results should be reported as in routine clinical practice. Two main platforms were used in the second testing wave in the collaborative study: PCR-based platforms and next-generation sequencing (NGS) platforms. The most utilized PCR-based platform in this collaborative study was the Cobas^®^ z480 (Roche Molecular Systems, Pleasanton, CA, USA), combined with the quantitative real-time Cobas^®^ EGFR mutation test v2 (Cobas) (Roche Molecular Systems, Pleasanton, CA, USA) method in eight laboratories; most of them used the Cobas^®^ cfDNA Sample preparation kit for cfDNA isolation (Roche Molecular Systems, Pleasanton, CA, USA) (n = 5), other kits used were the Maxwell^®^ RSC ccfDNA Plasma Kit (Promega, Madison, WI, USA) (n = 1), the Maxwell^®^ 16 FFPE Plus LEV DNA Purification Kit (Promega, Madison, WI, USA) (n = 1) and the QIAsymphony DSP Circulating DNA Kit (Qiagen, Hilden, Germany) (n = 1). The other PCR platforms used by the remaining laboratories were the following: Idylla^TM^ platform (Biocartis, Mechelen, Belgium) (n = 2), QuantStudio 5 Dx Real-Time PCR System (Thermo Fisher Scientific, Waltham, MA, USA) (n = 2), EasyPGX^®^ qPCR instrument 96 (Diatech Pharmacogenetics, Jesi, Italy) (n = 1), LightCycler^®^ 480 System (Roche Molecular Systems, Pleasanton, CA, USA) (n = 1), QX200 Droplet Digital PCR System (Bio-Rad, Hercules, CA, USA) (n = 1), Real-time PCR cycler–Rotor -Gene 3000A (n = 1) or shaking water bath (n = 1). The NGS methods were used by four laboratories and included the following platforms and methods for DNA analytics: Ion S5 system with the Oncomine™ Lung cfDNA Assay (Thermo Fisher Scientific, Waltham, MA, USA) (n = 2), GeneReader Platform with the QIAamp MinElute ccfDNA Kit (Qiagen, Hilden, Germany) (n = 1) or Nextseq 500 system with the Avenio ctDNA Targeted kit (Roche Molecular Systems, Pleasanton, CA, USA) (n = 1).

The results of the liquid biopsy testing from the different participating laboratories were received as expected. In the first wave, almost all laboratories detected sample No. 1 with the highest concentration of T790M. Only laboratory No. 7, which used Cobas/Entrogen for ctDNA-Isolation, reported the sample as negative (false negative). All laboratories correctly reported the negative sample (sample No. 2). Samples No. 3, 4, and 5, which contained different dilutions of HCC-8207 cell line DNA of T790M, were recognized by 8, 10, and 11 laboratories for samples No. 3, 4, and 5 respectively using either ddPCR, GeneReader, NGS, AmoyDx^®^ EGFR 29 Mutations Detection Kit (AmoyDx, Xiamen, China) or EasyPGX^®^ ready EGFR (Diatech Pharmacogenetics, Jesi, Italy). Four laboratories using Cobas v2 or Cobas/Entrogen had difficulties detecting T790M, even though there were differences in the sensitivity and specificity between these laboratories: laboratory No. 4 had only problems with one sample (sample No. 3), whereas the other centers had two (lab No. 6) or three (lab No. 9) invalid results (Table 4).

In the second testing wave of the test, two laboratories (lab No. 10 and 11) identified all samples correctly; most laboratories detected the activating mutations but not the T790M mutation (Table 5A). In general, sample No. 2 was challenging as it contained very low numbers of spiked DNA (5 T790M positive droplets). Sample No. 2 was found only by two centers (lab No. 10 and 11) which used the AmoyDx kit as the detection method and QX200 Droplet Digital PCR in combination with the QIAamp Circulating Nucleic Acid kit (Qiagen, Hilden, Germany) as the extraction method, respectively. In total, eight centers used the Cobas detection method (lab No. 4, 5, 6, 8, 18, 23, 24, and 25) (Table 5B). The centers which additionally used the Cobas^®^ extraction method detected all the requested samples as expected except sample No. 2. Laboratory No. 23, which used QIAsymphony DSP Circulating DNA Kit (Qiagen, Hilden, Germany) for extraction, could also report all samples apart from sample No. 2 correctly. Laboratory No. 18, which used the Maxwell^®^ RSC ccfDNA Plasma Extraction Kit had difficulties detecting samples No. 4, 7, and 9, with DNA levels at the detection limit of the Cobas^®^ method. Laboratory No. 24 used the Maxwell^®^ 16 FFPE Plus LEV DNA Purification Kit to extract DNA that was not developed for use in liquid biopsy. This lab could only report sample No. 1 correctly since, obviously, it was not possible to extract DNA from the samples sufficiently.

Among the four centers using the AmoyDx kit (lab No. 1, 7, 10, and 22), one laboratory utilized the Cobas^®^ cfDNA extraction sample preparation kit and classified all samples correctly (No. 10). The three other laboratories used AmoyDx^®^ Circulating DNA Kit (lab No. 1), QIAamp Circulating Nucleic Acid kit (lab No. 7) or MagNA Pure 24 Total NA Isolation Kit (Roche Molecular Systems, Pleasanton, CA, USA) for DNA extraction and detected all samples but one (sample No. 2) (Table 5C).

Four laboratories used an NGS platform (No. 16, 17, 19, and 21) (Table 5D); they interpreted the samples as expected-except for sample No. 2. Only laboratory No. 16, which used the Avenio ctDNA Targeted Kit and the Illumina platform NextSeq 500 System had difficulties in correctly detecting the dilutions and identified only three samples out of 10 (sample Nr. 1, 3, 6, and 8). An explanation might be found in the extraction step, as the DNA fragment size in the samples was bigger than normal ctDNA; this might have influenced the extraction process of these samples. This hypothesis is supported by the fact that the four correctly identified samples were the ones with the higher DNA concentration, as well as the negative samples.

Two centers (No. 3 and 20) used the detection and ctDNA extraction method from Idylla^TM^ ctEGFR Mutation Assay (Idylla). They showed a worse performance compared with the other methods used, as they detected four and seven samples out of a total of ten, respectively (Table 5E).

## 4. Discussion

This inter-laboratory comparison of ctDNA detection methods describes the performance of different PCR-based and NGS-based platforms for detecting T790M, del19, and L858R mutations in various laboratories across Central and Eastern Europe.

A first round (first testing wave) was performed to establish the collaborative study procedure. Centers that used the ddPCR detected all samples as expected, as well as centers using the AmoyDx kit, NGS, and EasyPGX^®^ ready EGFR. Centers using Cobas had difficulties in detecting various dilutions of T790M. One may consider that the highest dilution (sample No. 3) was below the threshold of this method. However, there were differences between the laboratories which used the Cobas system; laboratory No. 4 had only problems with one sample (sample No. 3), whereas the other centers had two (lab No. 6) or three (lab No. 9) invalid results.

In the second testing wave, most participating laboratories obtained the expected results using their in-house methods, despite differences in the platforms and the assay protocols used. Two centers, which used ddPCR and AmoyDx Kit, identified all samples correctly. Centers that used the Cobas extraction method combined with the Cobas ctDNA detection method identified all samples correctly, except the challenging sample No. 2, which contained, on average, only five positive droplets of T790M. This amount was below the threshold of the Cobas method. Interestingly, some centers detected the activating mutation L858R and del19, and others did not. One laboratory using the Cobas detection method in combination with Maxwell^®^ RSC ccfDNA Plasma Kit additionally did not detect samples No. 4, 7, and 9 containing low numbers of positive T790M/del 19 droplets, which were at the detection limit of the Cobas method. Another laboratory, which used the Cobas detection method in combination with the Maxwell^®^ 16 FFPE Plus LEV DNA Purification Kit, only detected sample No. 1 correctly. It is important to consider that the Maxwell^®^ 16 FFPE Plus LEV DNA Purification Kit was developed to extract DNA from tissue samples and not from a liquid biopsy. This procedure was chosen by the participating lab that routinely does not utilize liquid biopsy to verify if the extraction method established in-house would have the potential to be used in liquid biopsy. This result emphasizes the importance of using established and specific methods or procedures at any step of the analysis to get reliable results. Most of the laboratories using an NGS platform detected the samples as expected; only one laboratory (No. 16), which used the Avenio ctDNA Targeted Kit after extraction with Maxwell^®^ RSC ccfDNA Plasma Kit and the NextSeq 500 System, did not detect samples No. 2, 4, 5, 7, 9, and 10 containing a low number of positive T790M/del19 droplets. This might be due to the long DNA fragments, which may interfere with the method. Two laboratories, which used the detection and ctDNA extraction method from Idylla, showed a low performance as the results were not comparable with those obtained with the other detection methods analyzed. For one laboratory using Idylla, the volume of the sample (1 mL) was a problem; this was because Idylla requires 2 mL samples, and the laboratory had difficulties handling the samples. The laboratory divided the sample into two aliquots and tested them using two methods (Idylla and Cobas). This leads to an underperformance of the methods because the samples were then under the threshold for these methods; this might partly explain the poorer performance of Idylla.

Factors that may influence the accuracy of liquid biopsy are the pre-analytics, DNA analytics, and biological variability. For the pre-analytics, the type of blood tubes used is important; DNA can be stored at room temperature for several days using blood conserving tubes (BCTs), but samples should be processed within one or a maximum of two hours with EDTA tubes. Time and temperature between blood collection and processing of the plasma (BCTs should not be frozen during transport), centrifugation conditions, methods of quantification, and protocols used to isolate ctDNA are other critical factors in successfully performing a liquid biopsy. In this collaborative study, we did not control the pre-analytics, except for the protocol for ctDNA isolation. Robustness, analytical specificity and sensitivity of the test procedure, intrinsic errors in the PCR methodology, and technological sources of error are critical factors in DNA analytics. Regarding biological variability, the time point of sampling might as well influence the outcome of liquid biopsy.

Any method, either PCR- or NGS-based, can detect the T790M mutation. However, if a method with lower sensitivity is used, the result should be checked again in case of a negative result, either by repeating the liquid biopsy and/or performing an additional tissue biopsy. If the response to the treatment is no longer given or the patient’s condition deteriorates, the detection method should be repeated as soon as possible. If time is not a critical factor, the liquid biopsy can be repeated in an interval of two to three weeks.

In this collaborative study, ddPCR and AmoyDx detected all samples. Only these two methods identified the challenging sample No. 2 containing a very low concentration of spiked DNA with the T790M mutation (average number of five positive droplets). Laboratories using Cobas performed within the detection limit of the Cobas method; only sample No. 2 was below 25 copies/mL, which is under the detection limit of Cobas. Therefore, it was expected that most Cobas using laboratories should identify 90% of the samples, which was mainly the case. However, most of the samples in the clinical routine are under the threshold of 10 copies/mL, and this is possibly the reason why so many patients are not correctly identified and, therefore, cannot benefit from subsequent therapy in a timely manner. Previous data have shown a response rate of 61% in T790M-positive patients with ddPCR [6]. The response to osimertinib is independent of the copy number of the resistance mutations. However, recent data indicate that the copy number of the original activating mutation is important for a response to osimertinib [23].

The test had several limitations: we did not control the pre-analytics (except ctDNA extraction); moreover, DNA was not crushed into small fragments (<150 bp size), which might be a problem for some of the methods. There was only one sample with a low copy number (<10 copies/mL) available for testing.

The emergence of the acquired EGFR T790M resistance mutation after first- or second-generation EGFR-TKIs in EGFR-positive NSCLC has led to developing third-generation TKIs. A liquid biopsy is a powerful tool for identifying T790M mutations [21]. The correct re-characterization of patients, who have progressed on first- or second-generation TKIs and a reliable method to detect the T790M mutation, is key for initiating the subsequent second-line therapy and a favorable outcome for the patient.

## 5. Conclusions

EGFR testing using liquid biopsy may provide more therapy options for patients with EGFR-positive NSCLC. This is particularly important with respect to the sequential management of EGFR TKIs. The detection of T790M resistance mutations has crucial consequences on the subsequent second-line therapy with a third-generation EGFR-TKI such as osimertinib. As no global standards for testing T790M mutations in liquid biopsy currently exist, the present collaborative study describes the in-house liquid biopsy methods and their sensitivity against T790M, del19, and the L858R mutation. The results might offer an essential contribution to ensuring high-quality standards and contribute to developing a standard EGFR T790M testing in liquid biopsy.

## Figures and Tables

**Table 1:** Cell lines used for spiking the plasma in the second testing wave.

		Average Number of Positive Droplets			
Sample	Spiked DNA	EGFR_T790M_	EGFR_Del19_	EGFR_L858R_	EGFR_wt_
1	HCC-8207 (30 ng)	485	2279	0	+
2	CRL-5908 (0.25 ng)	5	0	7	+
3	A549 (50 ng)	0	0	0	+
4	HCC-8207 (1.5 ng)	34	139	0	+
5	HCC-8207 (3 ng)	49	465	0	+
6	CRL-5908 (100 ng)	780	0	951	+
7	HCC-8207 (1.5 ng)	25	132	0	+
8	MCF-7 (27 ng)	0	0	0	+
9	HCC-8207 (1.5 ng)	27	172	0	+
10	HCC-8207 (3 ng)	49	355	0	+

**Table 2:** Participating laboratories per country and their identification number (Lab No.) of the (**A**) and (**B**) testing waves.

Country	No. of Laboratories
(**A**)	
Austria	5
Bulgaria	1
Lithuania	1
Poland	4
Switzerland	1
	**12**
(**B**)	
Austria	9
Bulgaria	1
Croatia	1
Hungary	3
Lithuania	1
Poland	2
Russia	3
Slovakia	5
	**25**

**Table 3:** Extraction method (**A**) and detection method (**B**) used in the second testing wave of the collaborative study.

(**A**)	
**ctDNA Extraction Method**	**No. of Laboratories**
Cobas^®^ cfDNA Sample Preparation Kit	6
QIAamp Circulating Nucleic Acid Kit	4
Idylla^TM^ ctEGFR Mutation Assay	2
MagNA Pure 24 Total NA Isolation Kit	2
Maxwell^®^ RSC ccfDNA Plasma Kit	2
EZ1 ccfDNA Midi Kit	1
AmoyDx^®^ Circulating DNA Kit	1
QIAamp MinElute ccfDNA Kit	1
Maxwell^®^ 16 FFPE Plus LEV DNA Purification Kit	1
QIAsymphony DSP Circulating DNA Kit	1
(**B**)	
**Detection Method**	**No. of Laboratories**
Cobas^®^ EGFR Mutation Test v2	8
AmoyDx^®^ EGFR 29 Mutations Detection Kit	4
Idylla^TM^	2
Oncomine^TM^ Lung cfDNA Assay	2
Avenio ctDNA Targeted Kit	1
Droplet Digital PCR	1
EasyPGX^®^ ready EGFR	1
EGFR XL StripAssay^®^	1
QIAact Lung Plasma Track Panel	1

**Table 4:** Results from the first wave of the collaborative study. +: positive test; -: negative test; +-: inconclusive test. Numbers represent number of positive droplets.

Center	Assay	Sample 1	Sample 2	Sample 3	Sample 4	Sample 5
Reference	ddPCR	324	0	5	17	72
1	ddPCR	+	−	+	+	+
2	ddPCR	512	0	7.4	26.2	49.4
3	Genereader	+	−	+	+	+
4	Cobas v2	+	−	+	+	+
5	NGS	+	invalid	+	+	+
6	Cobas v2	+	invalid	invalid	invalid	+
7	Cobas/Entrogen	−	−	−	+	+
8	ddPCR	200	0	2	9	41
9	Cobas v2	+	invalid	invalid	invalid	invalid
10	AmoyDx	+	−	+	+	+
11/1	NGS	319	+-	8	22	65
11/2	ddPCR	+	−	+	+	+
12	EasyPGX	+	−	+	+	+

**Table 5:** Overview of the results from the second testing wave ring collaborative study.

**A**																										
**Sample**		**Center**																								
**No.**	**Name**	**1**	**2**	**3**	**4**	**5**	**6**	**7**	**8**	**9**	**10**	**11**	**12**	**13**	**14**	**15**	**16**	**17**	**18**	**19**	**20**	**21**	**22**	**23**	**24**	**25**
**1**	T790M/Del19																									
**2**	T790M/L858R																									
**3**	Wildtype																									
**4**	T790M/Del19																									
**5**	T790M/Del19																									
**6**	T790M/L858R																									
**7**	T790M/Del19																									
**8**	Wildtype																									
**9**	T790M/Del19																									
**10**	T790M/Del19																									
**Platforms**		**PCR**		**PCR**	**PCR**	**PCR**	**PCR**	**PCR**	**PCR**	**PCR**	**PCR**	**PCR**	**PCR**				**NGS**	**NGS**	**PCR**	**NGS**	**PCR**	**NGS**	**PCR**	**PCR**	**PCR**	**PCR**
**Detection kits**		**A**		**I**	**C**	**C**	**C**	**A**	**C**	**E**	**A**	**D**	**S**				**N**	**G**	**C**	**O**	**I**	**O**	**A**	**C**	**C**	**C**
**Extraction kits**		**A**		**I**	**C**	**C**	**C**	**Q^1^**	**C**	**Q^1^**	**C**	**Q^1^**	**Q^1^**				**M^1^**	**Q^2^**	**M^1^**	**E**	**I**	**R**	**R**	**Q^3^**	**M^2^**	**C**
**B**																										
**Sample**		**Center**																								
**No.**	**Name**	**4**	**5**	**6**	**8**	**18**	**23**	**24**	**25**																	
**1**	T790M/Del19																									
**2**	T790M/L858R																									
**3**	Wildtype																									
**4**	T790M/Del19																									
**5**	T790M/Del19																									
**6**	T790M/L858R																									
**7**	T790M/Del19																									
**8**	Wildtype																									
**9**	T790M/Del19																									
**10**	T790M/Del19																									
**Platforms**		**PCR**	**PCR**	**PCR**	**PCR**	**PCR**	**PCR**	**PCR**	**PCR**																	
**Detection kits**		**C**	**C**	**C**	**C**	**C**	**C**	**C**	**C**																	
**Extraction kits**		**C**	**C**	**C**	**C**	**M^1^**	**Q^3^**	**M^2^**	**C**																	
**C**																										
**Sample**		**Center**																								
**No.**	**Name**	**1**	**7**	**10**	**22**																					
**1**	T790M/Del19																									
**2**	T790M/L858R																									
**3**	Wildtype																									
**4**	T790M/Del19																									
**5**	T790M/Del19																									
**6**	T790M/L858R																									
**7**	T790M/Del19																									
**8**	Wildtype																									
**9**	T790M/Del19																									
**10**	T790M/Del19																									
**Platforms**		**PCR**	**PCR**	**PCR**	**PCR**																					
**Detection kits**		**A**	**A**	**A**	**A**																					
**Extraction kits**		**A**	**Q^1^**	**C**	**R**																					
**D**																										
**Sample**		**Center**																								
**No.**	**Name**	**16**	**17**	**19**	**21**																					
**1**	T790M/Del19																									
**2**	T790M/L858R																									
**3**	Wildtype																									
**4**	T790M/Del19																									
**5**	T790M/Del19																									
**6**	T790M/L858R																									
**7**	T790M/Del19																									
**8**	Wildtype																									
**9**	T790M/Del19																									
**10**	T790M/Del19																									
**Platforms**		**NGS**	**NGS**	**NGS**	**PCR**																					
**Detection kits**		**N**	**G**	**O**	**O**																					
**Extraction kits**		**M^1^**	**Q^2^**	**E**	**R**																					
**E**																										
**Sample**		**Center**																								
**No.**	**Name**	**3**	**20**																							
**1**	T790M/Del19																									
**2**	T790M/L858R																									
**3**	Wildtype																									
**4**	T790M/Del19																									
**5**	T790M/Del19																									
**6**	T790M/L858R																									
**7**	T790M/Del19																									
**8**	Wildtype																									
**9**	T790M/Del19																									
**10**	T790M/Del19																									
**Platforms**		**PCR**	**PCR**																							
**Detection kits**		**I**	**I**																							
**Extraction kits**		**I**	**I**																							

(A): all testing methods; (B) Cobas testing; (C) AmoyDx, (D) NGS, (E) Idylla^TM^. Abbreviations for detection kits: A: AmoyDx EGFR 29 Mutations Detection Kit; C: Cobas EGFR Mutation Test v2; D: ddPCR (digital droplet PCR); E: Easy PGX ready EGFR; G: QIAact Lung Plasma Track Panel; I: Idylla; N: AVENIO ctDNA Targeted Kit; O: Oncomine Lung cfDNA Assay; S: Shaking water bath. Abbreviations for ctDNA extraction kits: A: AmoyDx Circulating DNA Kit; C: Cobas; E: EZ1 ccfDNA Midi Kit; I: Idylla; M^1^: Maxwell RSC ccfDNA Plasma Kit; M^2^: Maxwell 16 FFPE Plus LEV DNA Purification Kit; Q^1^: QIAamp Circulating Nucleic Acid Kit; Q^2^: QIAamp MinElute ccfDNA Kit; Q^3^: QIAsymphony DSP Circulating DNA Kit; R: MagNA Pure 24 System. Green color: positive test; red color: negative test; dashed color: inconclusive test.

## Data Availability

Data available from the authors on request.

## Appendix A

Liquid biopsy collaborative study group—names and affiliations: Austria: Maximilian Prinz-Wohlgenannt, Gabriele Benetka, Institute for Clinical Pathology and Molecular Pathology, Hospital Horn, Austria; Martin Hyden, Elisabeth Schintler, Institute for Pathology, Hospital Klagenfurt/Wörthersee, Austria; Christian Paar, Christa Kubasta, Institute of Laboratory Medicine, Kepler University Hospital Linz, Austria; Christina Schuster, Elisabeth Muckenschnabel, Institute for Clinical Pathology and Molecular Pathology, Hospital Mistelbach, Austria; Selma Hönigschnabl, Edith Perkovits, Institute for Pathology and Bacteriology, Hospital Donaustadt, Vienna, Austria; Ulrike Setinek, Cornelia Aigner, Institute for Pathology and Microbiology, Hospital Ottakring, Vienna, Austria; Leonhard Müllauer, Felicitas Oberndorfer, Department of Pathology, Medical University of Vienna, Austria; Wolfgang Hulla, Tamara Kalchbrenner, Institute for Clinical Pathology and Molecular Pathology Thermenregion, Wiener Neustadt, Austria; Peter Obrist, Jasmin Mustedanagic, Tyrolpath Obrist Brunhuber GmbH, Molecular Pathology, Zams, Austria. Bulgaria: Alexey Savov, Stoyan Bichev, University Hospital “Maichin Dom”, National Genetics Laboratory, Sofia, Bulgaria. Croatia: Lovorka Batelja-Vuletic, Department of Pathology, University of Zagreb School of Medicine, Croatia and University Hospital Centre Zagreb, Croatia; Gordana Bubanovic, Laboratory for Molecular Pathology, Department of Pathology, University of Zagreb School of Medicine, Croatia and University Hospital Centre Zagreb, Croatia. Hungary: Erzsébet Csernák, Erika Tóth, National Institute of Oncology, Department of Surgical and Molecular Pathology, Budapest, Hungary; Csaba Bödör, Gergő Papp, Molecular Diagnostic Division, Department of Pathology and Experimental Cancer Research, Semmelweis University, Budapest, Hungary; Attila Mokánszki, Gábor Méhes, Department of Pathology, Faculty of Medicine, University of Debrecen, Hungary. Lithuania: Vaida Kutkienė, Dovilė Kalikauskienė Vabalienė, Hospital of Lithuanian University of Health Sciences Kauno Klinikos, Lithuania. Poland: Bartłomiej Budny, Elżbieta Wrotkowska, E.J. Zeyland Wielkopolska Center of Pulmonology and Thoracic Surgery, 60-569 Poznań and Department of Endocrinology, Metabolism and Internal Medicine, Poznan University of Medical Sciences, 60-355 Poznań, Poland; Andrzej Tysarowski, Cancer Molecular and Genetic Diagnostics Department and Department of Molecular and Translational Oncology, Maria Sklodowska-Curie National Research Institute of Oncology, Warsaw, Poland; Katarzyna Seliga, Department of Molecular and Translational Oncology, Maria Sklodowska-Curie National Research Institute of Oncology, Warsaw, Poland. Slovakia: Ľuboslav Mihók, Imrich Hikkel, National Cancer Institute, Department of Medical Genetics, Bratislava, Slovakia; František Gmitter, Lucia Fröhlichová, Luis Pasteur University Hospital Košice, Department of Pathology, Kosice, Slovakia; Jana Verebová, Slavka Kormaniková, Unilabs Slovakia s. r. o., Laboratory of Medical Genetics, Kosice, Slovakia; Anna Farkašová, Lukáš Plank, Martin’s Biopsy Center, Ltd., Martin, Slovakia.
